# Common Beans and Their Non-Digestible Fraction: Cancer Inhibitory Activity—An Overview

**DOI:** 10.3390/foods2030374

**Published:** 2013-08-02

**Authors:** Rocio Campos-Vega, B Dave Oomah, Guadalupe Loarca-Piña, Haydé Azeneth Vergara-Castañeda

**Affiliations:** 1 Food Graduate Program of Republic Center (PROPAC), Research and Graduate Studies in Food Science, School of Chemistry, Universidad Autónoma de Querétaro, Queretaro 76020, México; E-Mail: loarca@uaq.mx; 2 National Bioproducts and Bioprocesses Program, Pacific Agri-Food Research Centre, Agriculture and Agri-Food Canada, Summerland, BC, Canada V0H IZ0; E-Mail: oomahd@agr.gc.ca; 3 Nucitec, S. A. de C. V., Research Department, Comerciantes 15-3, Peñuelas, Queretaro 76148, México; E-Mail: hvergara@nucitec.com

**Keywords:** common beans, prevention, antitumor activities, non digestible fraction, bioactive compounds

## Abstract

The US Department of Agriculture’s MyPyramid guidelines introduced a near doubling of the dietary recommendations for vegetables including dry beans—an important food staple in many traditional diets that can improve public health and nutrition. Populations with high legume (peas, beans, lentils) consumption have a low risk of cancer and chronic degenerative diseases. Common beans (*Phaseolus vulgaris* L.) are known as a rich, reliable source of non-digested compounds like fiber, phenolics, peptides and phytochemicals that are associated with health benefits. Emerging evidence indicates that common bean consumption is associated with reduced cancer risk in human populations, inhibiting carcinogenesis in animal models and inducing cell cycle arrest and apoptosis in cell cultures. Fiber may reduce the risk of premature death from all causes, whereas the whole non-digestible fraction from common beans exhibits anti-proliferative activity and induces apoptosis *in vitro* and *in vivo* colon cancer. The mechanisms responsible for this apparently protective role may include gene-nutrient interactions and modulation of proteins’ expression. This review investigates the potential health benefits and bioactivity of beans on tumor inhibition, highlighting studies involving functional compounds, mainly non-digestible fractions that modulate genes and proteins, thereby, unraveling their preventive role against the development of cancer.

## 1. Introduction

Dry common bean (*Phaseolus vulgaris* L.) is a legume widely consumed throughout the world and is considered a good source of high protein (23%), complex carbohydrates, dietary fiber and some vitamins and minerals. The consumption of dry common beans has been associated with reduced risk of several chronic and degenerative diseases such as cancer, obesity, diabetes and cardiovascular diseases. In addition to the nutritional components, common beans are rich in many phytochemicals with potential health benefits such as polyphenolic compounds, fiber, lectins and trypsin inhibitors [1].

Carbohydrates constitute the main fraction of beans (55%–65% dry weight on average) with polysaccharides as the major constituents, and small but significant amounts of oligosaccharides (31%–76% of total sugars). Carbohydrate fraction of legumes include monosaccharides (ribose, glucose, galactose, and fructose), disaccharides (sucrose and maltose) the soluble sugar fraction, and oligosaccharides of the raffinose family (raffinose, stachyose, and verbascose), besides cellulose, lignin, pectin, galactose, arabinose, mucosa and xylose, that according to some authors, have to be grouped under the concept of “dietary fiber” or “non-digestible carbohydrates”. Dietary fiber, or cell wall material content in the cotyledon of legume seed is comparatively lower than that of the testa. The carbohydrate-oligosaccharide fraction of beans includes starch, soluble sugars and dietary fiber. Many health benefits are attributed to these components of bean seeds. The American Association of Cereal Chemists (AACC) defines dietary fiber (DF) as “the edible parts of plants or analogous carbohydrates that are resistant to digestion and absorption in the human small intestine with complete or partial fermentation in the large intestine” and it consists of polysaccharides (such as cellulose, hemicellulose and pectins), oligosaccharides, lignin and associated plant substances [2].

Recently, international authorities on dietary fiber definition, working through the CODEX Committee on Nutrition and Foods for Special Dietary Uses, have updated the terminology of the dietary fiber definition [3]. CODEX defines dietary fiber as carbohydrate polymers with 10 or more monomeric units, which are not hydrolyzed by the endogenous enzymes in the small intestine of humans and belong to the following categories: (1) edible carbohydrate polymers naturally occurring in the food as consumed; (2) carbohydrate polymers, which have been obtained from raw food material by physical, enzymatic or chemical means and which have a physiological beneficial effect on health as demonstrated by generally accepted scientific evidence to competent authorities; (3) synthetic carbohydrate polymers which have been shown to have a physiological effect of benefit to health as demonstrated by generally accepted scientific evidence to competent authorities. It is important to note that some problems regarding the partial measurement of resistant starch, polydextrose and resistant maltodextrins are encountered in the AOAC method. Most of the low molecular weight soluble dietary fiber (galactooligosaccharides, fructooligosaccharides, *etc.*) are not measured. Recently, a new integrated total dietary fiber procedure measuring all components, without duplication has been proposed [4].

Emerging evidence indicates that common bean consumption is associated with reduced cancer risk in human populations and rodent carcinogenesis models. Epidemiological and preclinical studies evaluating colon cancer [5,6,7,8] prostate cancer [9,10], mammary cancer [11] has lent further support for an inverse relationship between bean consumption and the development of cancer. However, the mechanism of action implicated in these health benefits is limited.

## 2. Common Bean and Non-Digestible Fraction

Colorectal cancer (CRC) is one of the most common neoplasms afflicting industrialized societies [12]. Both genetic and environmental exposures have been implicated in the etiology of CRC, and up to 75% of cases may be prevented by adequate/proper diets and regular exercise [13,14,15]. Furthermore, populations with high legume (beans, lentils (*Lens culinaris*), peas (*Pisum sativum*), peanuts (*Arachis hypogaea*) consumption have low risk of [8] and mortality from CRC [16].

Epidemiological evidence suggests a protective role of dietary fiber against CRC [17]. Several studies have shown the protective role of pulses, primarily due to the presence of phytochemicals such as phenolic compounds (condensed tannins, flavonoids, and anthocyanins), total dietary fiber (TDF) (soluble and insoluble), lectins, unsaturated fatty acids, phytic acid, trypsin inhibitors, and other secondary metabolites related to the prevention and/or reduction of chronic degenerative diseases [1,18,19,20]. Some of these components (non-digestible fraction (NDF) and phenolic compounds) may reach the colon to be fermented by the microflora, producing mainly short-chain fatty acids (SCFAs) such as acetic, propionic, and butyric acids [21]. The latter is a 4-carbon fatty acid that has been studied as a preventive agent because of its inhibition against tumor cell proliferation and induction of apoptosis leading to a more differentiated phenotype [22], thus reducing the risk of CRC. 

**Figure 1:**
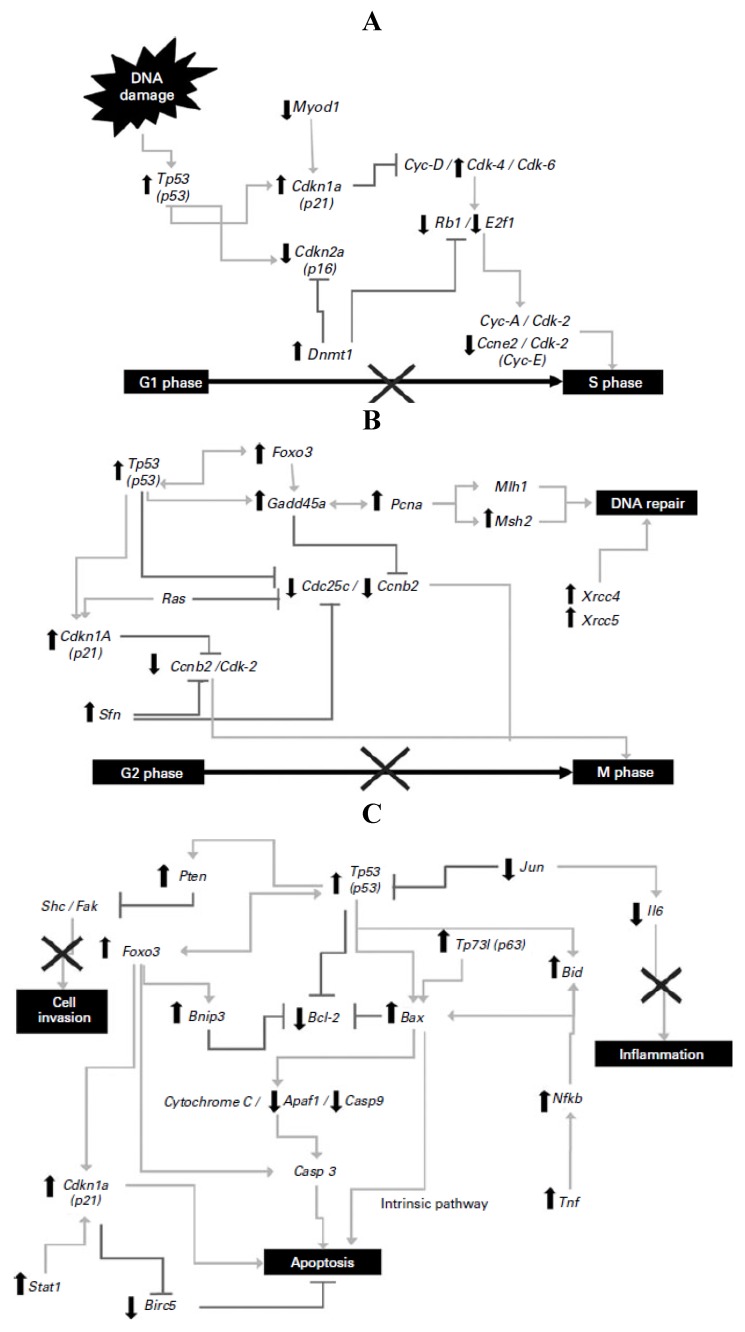
(**A**) Changes in gene expression in the G1/S cell-cycle phase; (**B**) changes in gene expression in the G2/M cell-cycle phase and DNA repair; (**C**) changes in gene expression in apoptosis and inflammatory pathways. Symbols indicate up-regulation (↑) and down-regulation (↓) in mRNA expression as derived from array analysis, and signaling pathway interruption (×). Note: this Figure is reproduced with permission from [25]. Copyright Vergara-Castañeda *et al.*, 2012.

Investigations in our laboratory [23,24,25] and by others [7,26,27,28] have demonstrated the potential of bean-based diets to inhibit azoxymethane (AOM)-induced colon cancer. For example, a polysaccharide extract of black bean cultivar (cv.) Negro 8025 reduced aberrant crypt foci development in azoxymethane-induced rats and regulated the expression of β-catenin, p53, p21, Rb, Bax and caspase-3 (Casp3) genes involved in cell proliferation, cellular arrest and apoptosis [23]. Cooked bean and NDF from Bayo Madero also provided direct protection against early stages of azoxymethane (AOM)-induced colon cancer in rats by suppressing aberrant crypt foci (ACF) and attenuating β-glucuronidase activity, enzymes with the ability to hydrolyze many glucuronide conjugates and thus, potentially releasing active carcinogenic metabolites in the intestinal lumen [24]. Furthermore, the transcriptional effects of the NDF from common bean cv. Bayo Madero on the gene expression profile in the distal colon tissue of Tp53 signal transduction in an *in vivo* model of early-stage colon cancer were investigated to elucidate the molecular mechanism involved in prevention [25]. Significant differences were detected in 72 genes of the Tp53-mediated signaling pathway involved in apoptosis, cell-cycle regulation and arrest, inhibition of proliferation and inflammation, and DNA repair illustrated in Figure 1. Tp53, Gadd45a, Cdkn1a and Bax were highly expressed (9.3-, 18.3-, 5.5- and 3.5-fold, respectively) in the NDF + AOM group, whereas Cdc25c, Ccne2, E2f1 and Bcl2 were significantly suppressed (9.2-, 2.6-, 18.4- and 3.5-fold, respectively), among other genes, compared with the AOM group, suggesting that prevention of aberrant crypt foci results from a combination of cell-cycle arrest in G1/S and G2/M phases and cell death by apoptotic induction.

**Figure 2:**
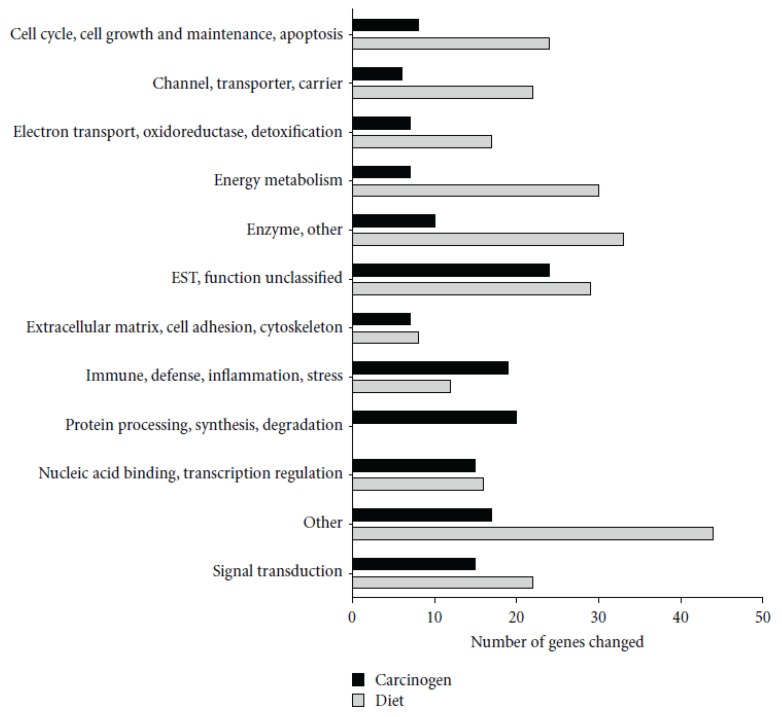
Functional classification of genes significantly altered by carcinogen azoxymethane (AOM) and by dietary treatment in the distal colon mucosa of rats detected by microarrays. A total of 155 genes were altered by carcinogen (AOM) and 257 by dietary treatment (Control *versus* BB *versus* SF, *p* < 0.05). Reproduced from Rondini and Bennink [28].

A research group has demonstrated that black bean (BB) and soy flour (SF)-based diets inhibit azoxymethane (AOM)-induced colon cancer and suggests beans inhibit colon carcinogenesis by modulating cellular kinetics and reducing inflammation, potentially by preserving mucosal barrier function [7,26,27,28]. Rondini and Bennink [28] showed that AOM treatment induced a number of genes involved in immunity, including several MHC II-associated antigens and innate defense genes (RatNP-3, Lyz2, Pla2g2a). Black bean- and soy flour-fed rats expressed genes involved in energy metabolism and water and sodium absorption and suppressed innate (RatNP-3, Pla2g2a, Tlr4, Dmbt1) and cell cycle-associated (Cdc2, Ccnb1, Top2a) genes. Genes involved in the extracellular matrix (Col1a1, Fn1) and innate immunity (RatNP-3, Pla2g2a) were induced by AOM in all diets, but to a lower extent in bean-fed animals (Figure 2).

The protection by common beans, NDF and SCFAs, has been studied using *in vitro* cell culture, widely used to assess the effect by which a substance induces differentiation and inhibits the survival of transformed cells. The HT-29 cell line has been used as a model to investigate the mechanism of some protective compounds [2,29]. In this regard, Campos-Vega *et al.* [30] demonstrated that common bean is an excellent source of NDF that can be fermented in the colon and produce SCFAs, compounds previously reported to exert health benefits (Table 1).

**Table 1:** Amount of short-chain fatty acids (SCFAs) (mmol/L) in fermented extract of non-digestible fraction (NDF) from cooked common bean seeds. Note: this table is reproduced with permission from [30]. Copyright Institute of Food Technologists, 2009.

	6 h			12 h			24 h		
Sample	Acetate	Propionate	Butyrate	Acetate	Propionate	Butyrate	Acetate	Propionate	Butyrate
*Negro 8025*	39 ± 0.3 ^Ax^	8 ± 0.0 ^Ay^	8 ± 0.3 ^Ay^	42 ± 1.0 ^Ax^	9 ± 0.6 ^By^	10 ± 0.6 ^Ay^	51 ± 0.7 ^Ax^	12 ± 0.7 ^By^	15 ± 0.7 ^Cz^
*Bayo Madero*	36 ± 0.3 ^Ax^	6 ± 0.6 ^Ay^	7 ± 0.3 ^Ay^	44 ± 0.3 ^Ax^	9 ± 0.7 ^By^	10 ± 0.6 ^Ay^	48 ± 1.0 ^Bx^	12 ± 0.3 ^By^	15 ± 0.3 ^Cz^
*Pinto Durango*	32 ± 0.7 ^Ax^	7 ± 0.3 ^Ay^	6 ± 0.0 ^Az^	36 ± 0.3 ^Bx^	9 ± 0.6 ^By^	8 ± 0.0 ^Bz^	48 ± 0.6 ^Bx^	9 ± 0.0 ^Cy^	13 ± 0.3 ^Cy^
*Azufrado Higuera*	33 ± 0.6 ^Ax^	8 ± 0.0 ^Ay^	6 ± 0.3 ^Ay^	37 ± 0.3 ^Bx^	11 ± 0.3 ^Ay^	9 ± 0.3 ^By^	39 ± 0.3 ^Cx^	14 ± 0.3 ^Ay^	14 ± 0.3 ^Bz^
*Rafinose (control)*	14 ± 0.5 ^By^	1 ± 0.1 ^Bx^	1 ± 0.1 ^Bx^	28 ± 2.7 ^Cy^	6 ± 1.0 ^Cx^	5 ± 0.7 ^Cx^	30 ± 0.6 ^Dy^	8 ± 0.4 ^Cx^	2 ± 0.2 ^Ax^

Results are the average of 3 independent experiments ± SEM; ^A*–*C^ Means in a column for varieties and SCFA with different letters are significantly different (*p <* 0.05); ^x*–*z^ Means in row for hours with different small letters are significantly different (*p <* 0.05).

**Figure 3:**
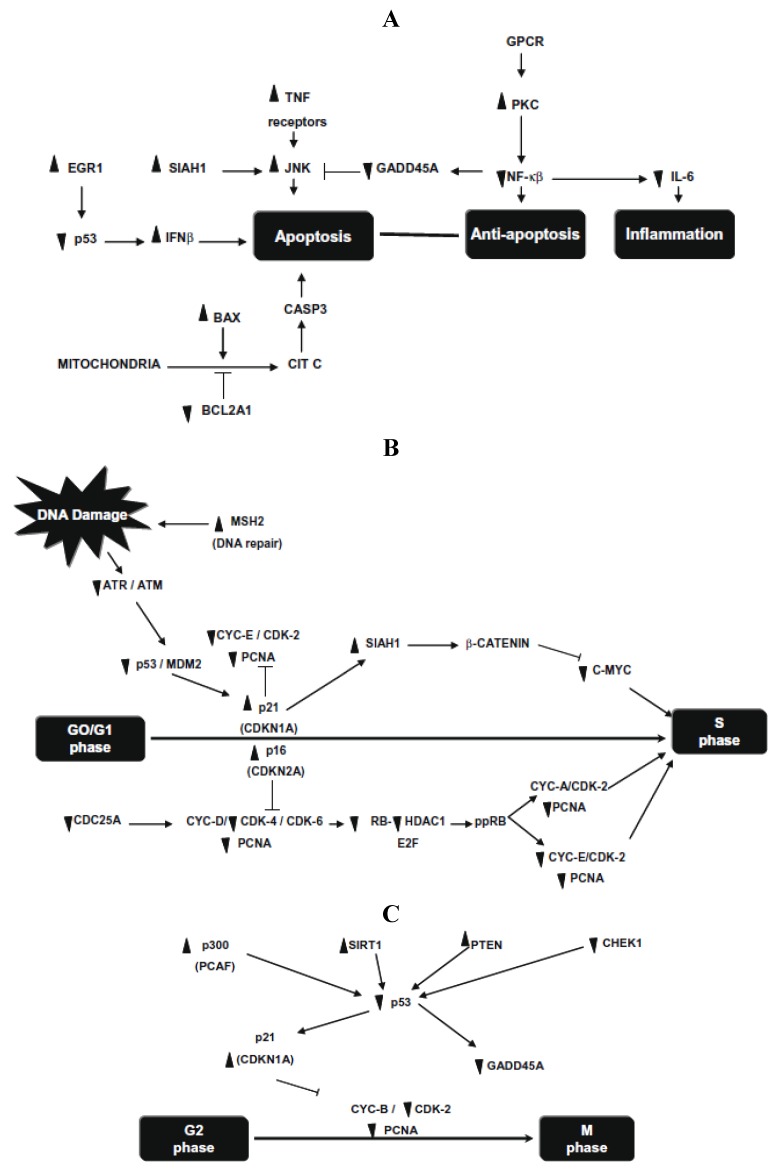
(**A**) Changes in gene expression in apoptosis and inflammatory pathways; (**B**) changes in gene expression in G1/S cell cycle phase; (**C**) changes in gene expression in G2/M cell cycle phase. Symbols indicate up-regulation (▲) or down regulation (▼) in mRNA expression as derived from array analysis. Note: this figure is reproduced with permission from [20]. Copyright Elsevier Ltd., 2010.

Later, this research group investigated the molecular changes of p53 pathway in HT-29 cells after 24 h exposure to *in vitro* (human gut flora (FE-hgf)) fermented NDF (cv. Bayo Madero) [20]. Significant differences were detected in 72 of 84 human p53-mediated signal transduction response genes involved in apoptosis, cell cycle and cell proliferation. Apoptosis genes, SIAH1, PRKCA, and negative regulation of the cell cycle gene MSH2, were the highest up-regulated genes (30.5-, 18.4- and 9.8-fold, respectively), whereas cell cycle genes, CHEK1 and GADD45A, were markedly down regulated (21.4- and 9.1-fold, respectively) (Figure 3). They demonstrate that common beans and/or its NDF modulate gene expression profiles in HT-29 cells, providing insight into the mechanism underlying its overall protective function against colon carcinogenesis.

**Figure 4:**
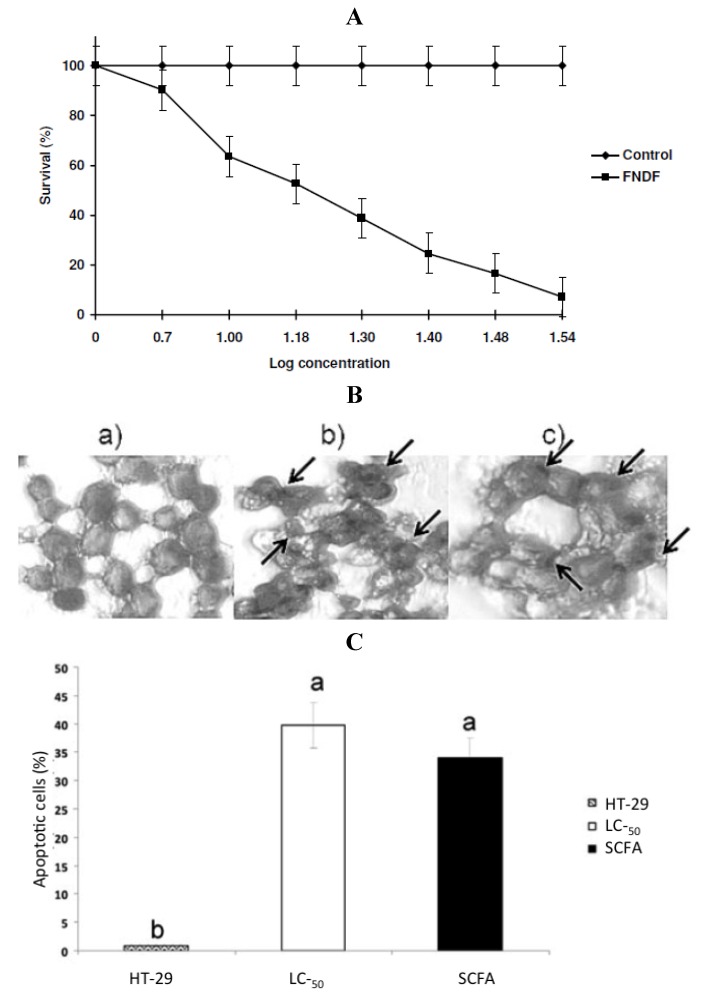
(**A**) Concentration-response curve of FNDF on HT-29 cells survival. Each value represents the average of 2 independent experiments ± SD. (**B**) Effect of FNDF on DNA fragmentation. Results are expressed as the percentage of apoptotic cells. (**B**) Apoptotic cells were identified by TUNEL technique DNA fragmentation that can be observed as brown spots (indicated by an arrow). (**a**) Control (untreated HT-29 cells); (**b**) LC_50_-FNDF treated cells; and (**c**) LC_50_-SCFA treated cells. (**C**) Apoptotic cells (%). Results are the mean ± standard error of 2 experiments with 2 repetitions each. Different letters by sample indicate significant difference α = 0.05, Tukey’s test. Note: this figure is reproduced with permission from [29]. Copyright Institute of Food Technologists, 2011.

Cruz-Bravo *et al.* [29] extended previous study with cultivar Negro 8025 [23] combining biochemical analysis with experiments designed to assess the effect of *in vitro* fermented NDF (FNDF) on the survival of the colon adenocarcinoma HT-29 cells. The results showed that FNDF inhibits HT-29 cell survival in a time and concentration-dependent manner (the lethal concentration 50 (LC_50_) was 13.63% FNDF (equivalent to 7.36, 0.33, and 3.31 mmol of acetic, propionic, and butyric acids, respectively)). DNA fragmentation, an apoptosis indicator, was detected by the TdT-mediated dUTP nick end labeling method (TUNEL) in cells treated with the LC_50_-FNDF and a synthetic mixture of SCFAs mimicking LC_50_-FNDF (Figure 4).

**Figure 5:**
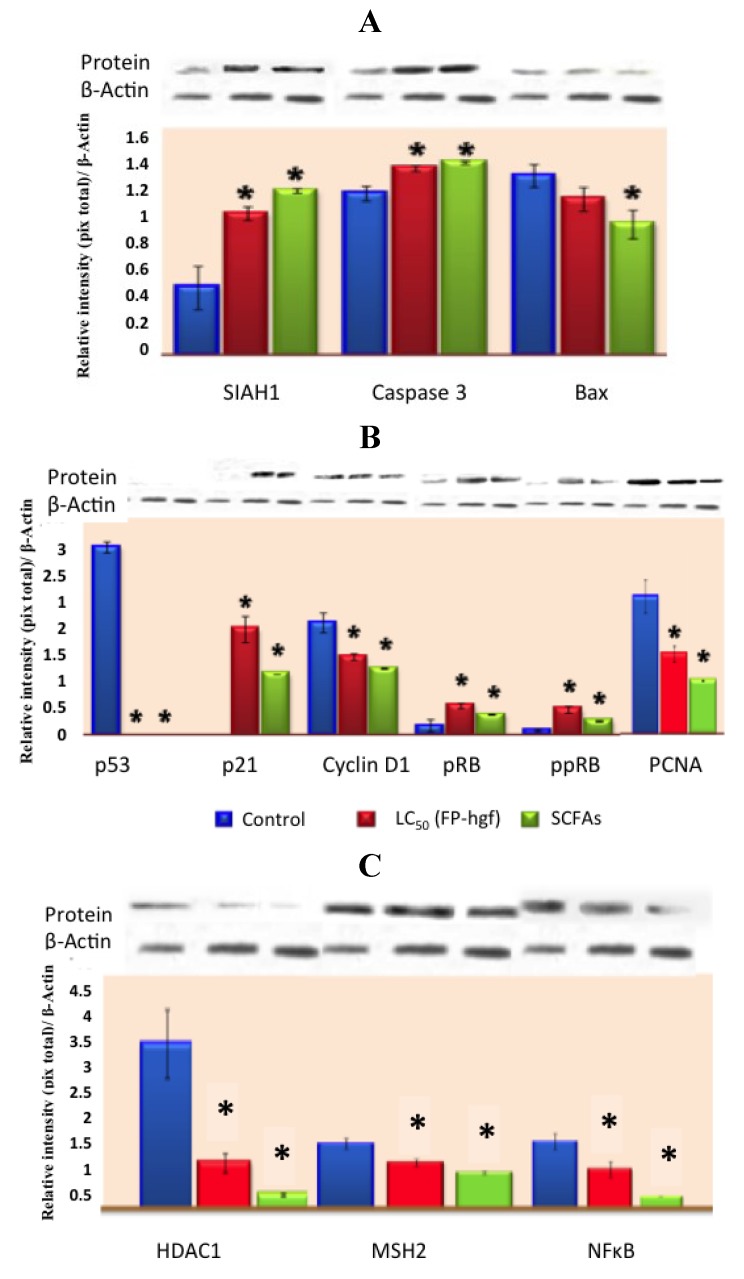
Expression of (**A**) apoptosis-related proteins, (**B**) cell cycle-related proteins and (**C**) expression of MSH2, NFκB, and HDAC1 related proteins in HT-29 cells after 24 h of treatment with LC_50_/FP-hgf and SCFAs mixture found in the LC_50_/FP-hgf. Expression was analyzed by Western blot using specific antibodies. Control: protein expression in cells without any treatment. The blot was tested with anti-actin antibody to confirm equal protein loading. The protein expression was normalized to β-actin. Data are the mean ± standard errors of three independent experiments (*p* < 0.05 *versus* control). Note: this figure is reproduced with permission from [31]. Copyright American Chemical Society, 2012.

Recently, Campos-Vega *et al.* [31], suggested that FNDF from common beans can elicit beneficial protective effects in colon cancer by modulating protein expression in HT-29 cells. FNDF inhibited HT-29 cell growth and modulated protein expression associated with apoptosis, cell cycle arrest, and proliferation (Figure 5), as well as morphological changes linked to apoptosis evaluated by TUNEL and hematoxylin and eosin stains, confirming previous results on gene expression. Meanwhile, a Korean kidney bean husk extract exhibited a series of antitumor effects such as cell death and apoptotic body appearance. These antitumor effects were accompanied by increases in p-AMPK (AMP-activated protein kinase, possible target molecule of tumor control) and p-Acc, as well as antitumor proteins p53 and p21 [32].

**Figure 6:**
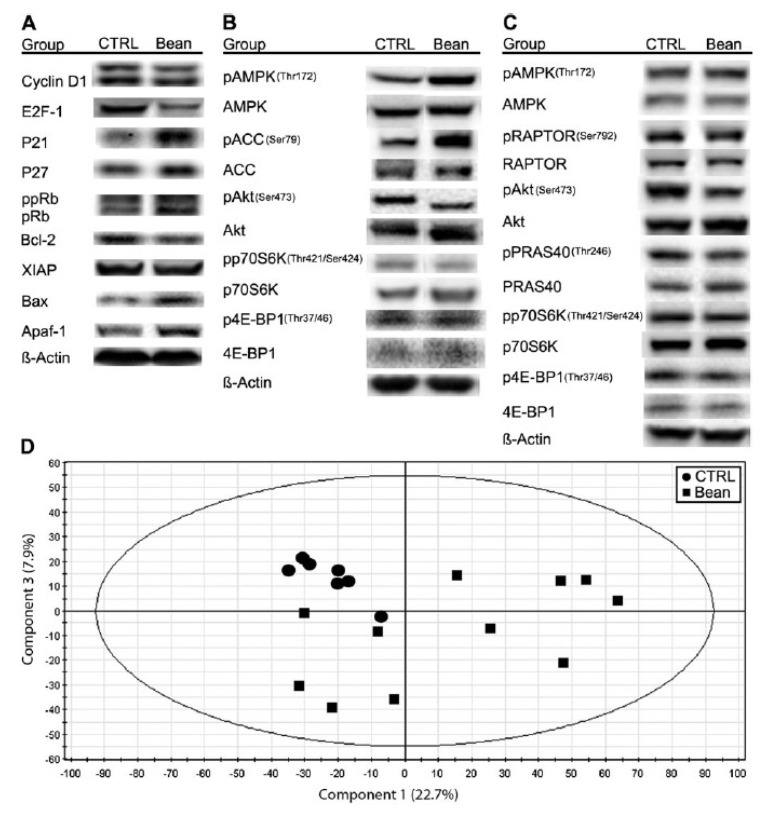
Effects of bean feeding on cell cycle and apoptosis regulators, mTOR signaling and the plasma metabolome. The images were directly acquired from the ChemiDoc work station equipped with a CCD camera (1300 × 1030 resolution). (**A**) A composite image of representative western blots of lysates of carcinomas from control (CTRL) and bean-fed (bean) rats. Images are for cell cycle regulators, cyclin D1, E2F-1, p21Cip1, p27Kip1 and Rb (ppRb, hyper-phosphorylated Rb; pRb, hypo-phosphorylated Rb) and apoptosis regulators, Bcl-2, X-linked inhibitor of apoptosis protein (XIAP), Bax and Apaf-1. (**B**) A composite image of representative western blots of lysates of carcinomas from control (CTRL) and bean-fed (bean) rats. Images are for components of the AMPK-Akt-mTOR signaling network, phosphorylated and total: AMPK, ACC, Akt, p70S6 kinase (p70S6K) and 4E-binding protein 1 (4E-BP1). (**C**) A composite image of representative western blots of lysates of liver from control (CTRL) and bean-fed (bean) rats. Images are for phosphorylated and total: AMPK, Raptor, Akt, PRAS40, p70S6K and 4E-BP1. (**D**) Scores scatter plot from principal component analysis (PCA) of plasma demonstrating the separation between rats fed control diet (circles) and bean diet (60% wt/wt) (squares). (control, *n* = 57; bean fed, *n* = 51). Note: this figure is reproduced with permission from [11]. Copyright Thompson *et al.*, 2012.

In the Four-Corners Breast Cancer Study, bean consumption was related to reduced breast cancer risk; Hispanic women consuming a native Mexican diet (characterized by higher pulse consumption, such as common bean) had two-thirds the breast cancer incidence of the non-Hispanic white population whose diet was characterized as high in red meat, sugar and processed foods [33]. Common beans significantly inhibited the post-initiation stage of chemically induced mammary carcinogenesis in rats [34]. The investigations of dietary bean consumption have centered on: (1) systemic factors (e.g., glucose-dependent growth factor signaling, inflammatory pathways); (2) cell autonomous mechanisms (e.g., cellular energy and nutrient-sensing networks) such as the mammalian target of rapamycin (mTOR) network and (3) signaling pathways through which systemic factors regulate cell proliferation and apoptosis. The emerging evidence shows that the mTOR network is deregulated in cardiovascular disease, type-2 diabetes, and cancer, including breast cancer [35,36,37,38]. Recently, the cancer-associated molecular targets mediating the effects of bean on cancer burden were identified in a chemically induced rat model of breast cancer [11]. Beans reduced the carcinoma burden (62%, *p* < 0.001), particularly where the dominant cellular process was associated with apoptotic induction. The observed changes are related with phosphorylation status of mammalian target of rapamycin (mTOR) substrates (4E-binding protein 1 and p70S6 kinase), mTOR regulators adenosine monophosphate-activated protein kinase and protein kinase B (Akt) (p < 0.001); a reduced mTOR network activity in the liver is associated with an altered lipid metabolism (Figure 6). Identification of a role for the mTOR signaling network in the reduction of cancer burden by dietary bean is highly relevant given that this pathway is deregulated in most human breast cancers. 

Chromatography-time-of-flight MS identified candidate metabolic processes that account for dry bean effects on disease risk with a specific focus on the development of breast cancer [39]. Principal component analysis (PCA) of mass spectral data reveals that tissue of both types from control-fed *v.* bean-fed rats could be distinguished by their metabolomic profiles. Candidate ion identification using MassTRIX analysis software reveals that alterations in eicosanoid, fatty acid, TAG and steroid metabolism partially accounted for the differences observed in both PCA. In addition, results were consistent with the hypothesis that the varying inhibitory effects of genetically distinct dry bean types were mirrored by different patterns of lipid metabolites in mammary carcinoma. The use of MassTRIX provided links for metabolite profile enrichment with metabolic pathways in the *Kyoto Encyclopedia of Genes and Genomes*. Implicated pathways included a linkage between diacylglycerol and protein kinase C and eicosanoid metabolites and inducible cyclo-oxygenase-2 and/or eicosanoid degradation mediated via 15-PG dehydrogenase. These pathways have been reported to be misregulated during cancer development. The differences observed between control-fed and bean-fed rats in lipid metabolism require validation using targeted analytical methods and detailed analyses of how bean bioactive food components regulate genes that control lipid biosynthesis, inter-conversion and catabolism.

Although no evidence is available for the mechanism of action of common beans on other kinds of cancer, the antiproliferative effect of legumes, including Adzuki beans (*Vigna angularis*), has been explored [40]. Adzuki bean exhibited the strongest antiproliferative properties in a dose-dependent manner against all digestive system cancer cell lines (CAL27, AGS, HepG2, SW480 and Caco-2), ovary cancer cell SK-OV-3 and breast cancer cell MCF-7 among all legumes tested. A semi-pure protein fraction, containing the Bowman-Birk-type protease inhibitor from Tepary bean (*Phaseolus acutifolius*) seeds, showed differential cytotoxic effect, as well as an increase in cell attachment to culture dishes when tested for its *in vitro* effect on transformed cells [41]. It was responsible for the increase in cell adhesion, decreasing culture dishes’ extracellular matrix degradation, leading to a decrease of the *in vitro* cell invasion capacity. This effect coincided with the suppression of Matrix Metalloproteinase-9 activity indicating that Tepary bean seeds contain at least two different groups of bioactive proteins with distinct effects on cancer cells. Nakaya *et al.* [42] suggested that adzuki bean and its heat-stable extract is immunopotentiating foods that can be used as dietary supplements for cancer prevention and immunotherapy. Adzuki bean stimulates differentiation of bone marrow cells into immature dendritic cells with the greatest efficacy compared to 30 types of edible beans with biological activity. The level of IL-6 produced by sequential treatment of dendritic cells with Adzuki extract and lipopolysaccharide was the highest among the examined beans. Adzuki extract also inhibited the growth of human leukemia U937 cells, leading to induction of apoptosis.

## 3. Other Compounds

**Figure 7:**
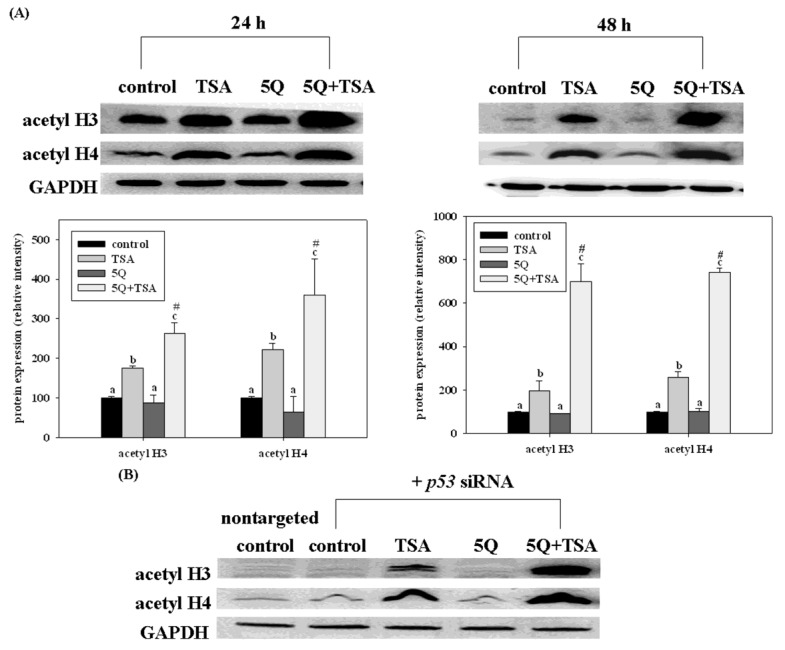
Effects of trichostatin A (TSA) alone or in combination with quercetin on the expression of acetyl histone H3 (acetyl H3) and H4 (acetyl H4) in A549 cells without (**A**) or with (**B**) p53 siRNA transfection. The cells were incubated with TSA (25 ng/mL) alone or in combination with 5 mM quercetin (5Q) for 24 or 48 h. Values (means 6 SD, *n* = 3) not sharing a common letter (**a**–**c**) are significantly different (*p* = 0.05). ^#^ Denotes a significant interaction between TSA and quercetin (two-way ANOVA, *p* = 0.05). Reproduced from Chan *et al.* [46].

Common beans are an excellent source of nutraceutical constituents such as fiber, protease inhibitors, phytic acid, and polyphenols such as tannins [43]. These compounds have antioxidant, antimutagenic, and anticarcinogenic activities and also scavenge free radicals [1,44,45]. Recently, quercetin, a major bean flavonoid was evaluated for its anti-tumor effect on trichostatin A (TSA), a novel anticancer drug in human lung cancer [46]. Quercetin significantly increased the growth arrest and apoptosis in A549 cells (expressing wild-type p53). However, such enhancing effects of quercetin were not significant in TSA-exposed H1299 cells (a p53 null mutant). Transfection of p53 siRNA into A549 cells significantly but not completely diminishes the enhancing effects of quercetin on TSA-induced apoptosis. Furthermore, quercetin enhanced TSA-induced apoptosis through the mitochondrial pathway. Transfection of p53 siRNA abolished such enhancing effects of quercetin. However, quercetin increased the acetylation of histones H3 and H4 induced by TSA in A549 cells, even with p53 siRNA transfection as well as in H1299 cells (Figure 7). In a xenograft mouse model of lung cancer, quercetin enhanced the antitumor effect of TSA. Tumors from mice treated with TSA in combination with quercetin had higher p53 and apoptosis levels than those from control and TSA-treated mice. These data indicate that regulation of the expression of p53 by quercetin plays an important role in enhancing TSA-induced apoptosis in A549 cells. However, p53-independent mechanisms may also contribute to the enhancing effect of quercetin.

Choi and Kim [47] investigated the antiproliferative activity of the isoflavones daidzein and genistein of common beans [48] in three breast cancer cell lines with different patterns of estrogen receptor (ER) and c-erbB-2 protein expression (ERα‑positive MCF-7 cells, c-erbB-2-positive SK-BR-3 cells and ERα/c-erbB-2-positive ZR-75-1). After treatment at various concentrations (1–200 µM for 72 h), the effect of daidzein and genistein on the proliferation of different cell types varied; these effects were associated with ERα and c-erbB-2 expression. Ferulic acid, the most abundant phenolic acid in common beans [48], when combined with 2-deoxy-d-glucose (2DG) along with irradiation, has been suggested for their oxidative mechanism of action on NCI-H460 (non-small cell lung carcinoma) cells involving alteration in p53, p21, NF-κB, Bax, and caspase-3 expression [49]. Additionally, Prabhakar *et al.* [50] explored the anti-cell proliferative efficacy of ferulic acid by analyzing the expression pattern of cell proliferative markers, proliferating cellular nuclear antigen (PCNA) and cyclin D1, in the buccal mucosa of golden Syrian hamsters treated with 7,12-dimethylbenz(a)anthracene (DMBA). Immunohistochemical (PCNA) and RT-PCR (Cyclin D1) analysis revealed over expression of PCNA and cyclin D1 in the buccal mucosa of hamsters treated with DMBA alone (tumor bearing hamsters). Oral administration of ferulic acid at 40 mg/kg body weight to hamsters treated with DMBA not only completely prevented the tumor formation, but also suppressed PCNA and cyclin D1 expression. This result suggests that ferulic acid may inhibit tumor formation in the buccal mucosa of hamsters treated with DMBA through its anti-cell proliferative potential as evidenced by decreased PCNA and cyclin D1 expression.

It has been demonstrated that anthocyanin-rich extracts from berries and grapes, and several pure anthocyanins and anthocyanidins exhibit pro-apoptotic effects in multiple cell types such as colon, breast, prostate, and leukemia cancer cells [51]. The main anthocyanins identified in seed coats of beans are delphinidin 3-glucoside (65.7%), petunidin 3-glucoside (24.3%), and maldivin 3-glucoside (8.7%) [2]. Anthocyanins induce apoptosis through both intrinsic (mitochondrial) and extrinsic (Fas) pathways. In the intrinsic pathway, the treatment of cancer cells with anthocyanin results in destabilization of the mitochondrial membrane, cytochrome *c* release and activation of caspase-9, and -3 as well as pro-apoptotic protein such as apoptosis inducing factor. In the extrinsic pathway, anthocyanins modulate the expression of Fas and FasL (Fas ligand) in cancer cells, that activates caspase-8, then cleaves Bid to tBid, and ultimately stimulates cytochrome *c* release [51]. Furthermore, structure-activity studies suggest that the potency of epidermal growth factor receptor (EGFR) inhibitors, a target of an expanding class of anticancer therapies, may be positively correlated with the presence of hydroxyl functions in the 3′ and 5′ positions of ring B of the anthocyanidin molecule, and inversely with the presence of methoxy groups in these positions. These findings provide important molecular basis for the antitumor properties of anthocyanidins [51].

On the other hand, riboflavin—a water-soluble vitamin—is a precursor of flavin mononucleotide (FMN) and flavin adenine dinucleotide (FAD), participating in various redox reactions essential to aerobic cell function. Beans are sources of riboflavin [2]. Therefore, riboflavin deficiency in blood is presumed to be related to a disturbance in riboflavin absorption. Disturbances in the steps in intermediary metabolism may occur if riboflavin intake is inadequate. Suppression of riboflavin transporter 2 (RFT2) mRNA and protein were closely related to the progression of gastric cancer (GC) lesions and RFT2 protein expression was positively associated with blood riboflavin levels and development of GC [52]. The *RFT2* gene may be the key target of environmental and genetic factors in the development of GC. Zinc and iron are present in a variety of beans in high quantities and phytosterols in small quantities [2]. Zinc deficiency (ZD) increases the risk of esophageal squamous cell carcinoma. In a rat model, chronic ZD induced inflammatory gene signature that fuels ESCC development, microRNAs regulated gene expression and are aberrantly expressed in cancers. Alder *et al.* [53] investigated whether chronic ZD (23 weeks) also induces a protumorigenic microRNA signature. Using the nanoString technology, they evaluated microRNA profiles in ZD esophagus and six additional tissues (skin, lung, pancreas, liver, prostate and peripheral blood mononuclear cells). ZD up-regulated inflammation genes and altered microRNA expression (dysregulation of *miR-31* and *miR-21*) across all tissues, predictive of disease development. Iron deficiency accelerates Helicobacter Pylori-induced carcinogenesis in rodents and humans [54]. Several studies on cell cultures and xenograft mouse models suggest that dietary phytosterols may also exert protective effects against common cancers. Llaverias *et al.* [55] examined the effects of a dietary phytosterol supplement on tumor onset and progression using the well-characterized mouse mammary. Both the development of mammary hyperplastic lesions (at 4 weeks) and total tumor burden (at 13 weeks) were reduced after dietary phytosterol supplementation in female mice fed a high-fat, high-cholesterol diet. A blind, detailed histopathological examination of the mammary glands (at 8 weeks) also revealed the presence of less-advanced lesions in phytosterol-supplemented mice. This study provides preclinical proof of concept that dietary phytosterols could prevent the tumor growth associated with fat-rich diet consumption. 

## 4. Conclusions

Dietary modification by increasing the daily consumption of a wide variety of common beans is a practical strategy for consumers to optimize their health and reduce the risk of cancer. Beans are good sources of bioactive compounds and recent evidence provides information of their impact and mechanism of action on this pathology, mainly for non-digestible fraction of beans on colon cancer. This effect seems to be related mainly to cell proliferation, cell cycle, apoptosis, cell invasion and histone modulation. Further research is warranted regarding the implications and the molecular mechanisms in which common beans and their bioactive compounds modulate the development of different types of cancer.
